# The prognostic value of m6A-related LncRNAs in patients with HNSCC: bioinformatics analysis of TCGA database

**DOI:** 10.1038/s41598-021-04591-z

**Published:** 2022-01-12

**Authors:** Liu-qing Zhou, Jin-xiong Shen, Jie-yu Zhou, Yao Hu, Hong-jun Xiao

**Affiliations:** 1 Department of Otorhinolaryngology, Union Hospital, Tongji Medical College, Huazhong University of Science and Technology, Wuhan, 430022 China; 2 Department of Otolaryngology-Head and Neck Surgery, Shanghai Ninth People’s Hospital, Shanghai Jiaotong University School of Medicine, Shanghai, 200011 China; 3 Department of Otorhinolaryngology, The Central Hospital of Wuhan, Huazhong University of Science and Technology, Wuhan, 430000 China

**Keywords:** Cancer, Diseases, Oncology

## Abstract

N6-methyladenosine (m6A) modifications play an essential role in tumorigenesis. These modifications modulate RNAs, including mRNAs and lncRNAs. However, the prognostic role of m6A-related lncRNAs in head and neck squamous cell carcinoma (HNSCC) is poorly understood. Based on LASSO Cox regression, enrichment analysis, univariate and multivariate Cox regression analysis, a prognostic risk model, and consensus clustering analysis, we analyzed 12 m6A-related lncRNAs in HNSCC sample data from The Cancer Genome Atlas (TCGA) database. We found 12 m6A-related lncRNAs in the training cohort and validated them in all cohorts by Kaplan–Meier and Cox regression analyses, revealing their independent prognostic value in HNSCC. Moreover, ROC analysis was conducted, confirming the strong predictive ability of this signature for HNSCC survival. GSEA and detailed immune infiltration analyses revealed specific pathways associated with m6A-related lncRNAs. In this study, a novel risk model including twelve genes (SAP30L-AS1, AC022098.1, LINC01475, AC090587.2, AC008115.3, AC015911.3, AL122035.2, AC010226.1, AL513190.1, ZNF32-AS1, AL035587.1 and AL031716.1) was built. It could accurately predict HNSCC outcomes and could provide new therapeutic targets for HNSCC patients.

## Introduction

Head and neck squamous cell carcinoma (HNSCC) is a common cancer in humans and is the fifth most common cancer in the world. It represents approximately 3% of new cancer cases and accounts for 2% of all cancer deaths annually worldwide^1^. Current treatment strategies for patients with HNSCC involve surgery, chemotherapy, immunotherapy, radiotherapy and so on. However, with such treatments, the survival results of HNSCC patients are still not satisfactory^2^. This poor prognosis is associated with local invasion and cervical lymph node metastases; additionally, a high rate of chemoresistance and locoregional recurrence has been noted among HNSCC patients^3,4^. It has been reported that the most important prognostic factor for patients with HNSCC is the clinical stage of disease (TNM stage); however, survival is variable for patients with the same stage, and more prognostic markers are needed^5^. It is urgent to search for new prognostic biomarkers and therapeutic targets to overcome the low HNSCC survival rates^6,7^.

N6-methyladenosine (m6A) was first reported in 1974, and m6A modification is the most abundant internal modification in eukaryotes^8^. m6A modification is a reversible process involving m6A regulators, including (“readers”) m6A-binding proteins, (“writers”) adenosine methyltransferases and (“erasers”) m6A demethylating enzymes^9^. Previous studies have revealed that m6A modification plays key roles in oncogenesis and tumor progression in several kinds of cancers, including lung cancer, breast cancer, acute myeloid leukemia and HNSCC^10^.

LncRNAs are RNAs greater than 200 nucleotides (nt) in length that possess many structural features of mRNAs without conserved open reading domains^11^. Many studies have illustrated that aberrantly expressed lncRNAs have complex and wide-ranging functions in tumors, including HNSCC^12,13^. While m6A modifications are known to modulate RNAs, including mRNAs, tRNAs, snRNAs and lncRNAs^14^. Despite the fact that m6A has been implicated in the occurrence and progression of various cancers, m6A RNA modification has not been extensively studied in HNSCC. LncRNA‐activating regulator of DKK1 is stabilized by m6A methylation and promotes cancer progression via forming a ternary complex in HNSCC^15^. m6A-binding proteins have complex regulatory mechanisms in nasopharyngeal carcinoma although their precise effects were rarely known^16,17^. Indeed, the precise mechanisms underlying m6A RNA modification in HNSCC are even less well understood. In the future, it is necessary to further clarify the key role of m6A in head and neck tumors, understand the mechanism of tumor occurrence and development comprehensively, and find more effective intervention measures and treatment approaches.

In this study, RNA sequencing data from the cancer genome map of The Cancer Genome Atlas (TCGA) dataset were used to estimate the effect of m6A-related lncRNAs on the prognosis of HNSCC patients. We constructed a risk signature by selecting twelve m6A-related lncRNAs after Cox univariate analysis and least absolute shrinkage and selection operator (LASSO) Cox regression analysis. Then, the prognostic role of the risk signature in HNSCC patients was analyzed. Moreover, we used the constructed signature to explore the association between the immune microenvironment and m6A-related lncRNAs. Our study aimed to comprehensively assess the correlation of m6A-related lncRNAs with the immune microenvironment, prognosis and therapeutic efficacy in HNSCC patients.

## Methods

### Patients and samples

The clinical characteristics of enrolled samples were also extracted from the TCGA-HNSCC database. 511 HNSCC patients were enrolled in the study.The collected clinicopathological data included sex, age, TNM classification, survival status, stage and survival outcomes. 310 patients were alive while 201 patients were dead. The number of patients aged ≤ 65 years was 336, 175 patients were > 65 years old. 137 patients were female and 374 patients were male. The number of stage I, II were 102 and the number of stage III, IV were 337, the stage of 72 patients were unknown. The number of grade I, II were 363 and the number of grade III, IV were 125, the grade of 23 patients were unknown. The number of T0, T1, T2 were 190 and the number of T3, T4 were 260, 61 patients were unknown. The number of M0 were 184 and the number of M1 were 1, 326 patients were unknown. The number of N0, N1 were 239 and the number of N2, N3 were 175, 97 patients were unknown.

### RNA sequence analysis and m6A-related genes

RNA-sequence (42 normal and 487 tumor) data of 511 patients was extracted from the TCGA-HNSCC database. Differentially expressed lncRNAs between HNSCC and normal tissues were determined by R software (R, A Language and Environment for Statistical Computing, R Core Team, R Foundation for Statistical Computing, Vienna, Austria. 2021, URL http://www.R-project.org, R version 4.0.5). According to previous publications, expression matrixes of 21 m6A-related genes were extracted from the TCGA databases, including expression data on writers (METTL3, METTL14, METTL16, WTAP, VIRMA [KIA1499], RBM15, RBM15B, and ZC3H13), erasers (FTO and ALKBH5) and readers (YTHDC1, YTHDC2, IGF2BP1, IGF2BP2, IGF2BP3, YTHDF1, YTHDF2, YTHDF3, HNRNPC, HNRNPA2B1, and RBMX)^18,19^.

### Immune infiltration analyses

We used the R software package Consensus Cluster Plus (CCP) to perform tumor cluster classification. "CIBERSORT" and "ESTIMATE" R package were used to detect tumor-infiltrating immune cells and compare the level of microenvironment scores between the two clusters. The ESTIMATE algorithm was applied to infer the levels of cell immune responses and estimate the tumor purity in tumor samples between two clusters based on the m6A lncRNA signature^20^. The algorithm is based on single-sample gene set enrichment analysis (GSEA) and generates three scores: (1) stromal score (the presence of matrix in tumor tissue); (2) estimate score (the inference of tumor purity); and (3) immune score (the infiltration of immune cells in tumor tissue). Furthermore, CIBERSORT was used to assess the immune cellular components and estimate the immune cell composition in the sample^21^. The immune cellular distributions of each HNSCC sample are presented by the barplot package. The differential proportions of 22 immune cells between two clusters were visualized via the vioplot package in R software.

### Development of the m6A-related lncRNA prognostic signature

Kaplan–Meier and univariate Cox regression analyses were conducted using the “survival” R package in the training cohort to screen for potential prognostic genes. Only genes that showed significance (p values < 0.05) in both Kaplan–Meier and Cox analyses were considered potential prognostic genes. The potential prognostic m6A-related genes were identified as overlapping potential prognostic genes and m6A-related genes, which were then entered into an overall survival-based LASSO Cox regression model in the training cohort. The LASSO analysis with twelve cross-validations was conducted by applying the “glmnet” R package, with the best penalty parameter lambda. A prognostic gene list with coefficients was generated from the LASSO model by the optimal lambda value. As shown in the following formula, each patient’s risk score was obtained from the gene expression levels and the corresponding coefficients. We developed a m6A-related lncRNA prognostic signature for the HNSCC patients involving twelve m6Arelated lncRNAs. $Risk\;score = \sum\nolimits_{i = 1}^{n} {Coef_{i} *x_{i} }$, C*oefi* means coefficients, and *xi* is the FPKM value of each m6A-related lncRNA. Patients in the training cohort were divided into low- and high-risk groups by the cutoff value of the median risk score. Kaplan–Meier analysis was used to measure the survival difference between the two groups. The prognostic ability of the gene signature was further assessed by Cox and ROC analyses. The prognostic capacity of the gene signature in the validation cohorts was validated by adopting the same formula and statistical methods. Gene set enrichment analysis (GSEA) was also performed to define the m6A-related lncRNA signatures in the KEGG database, which were then searched in the TCGA-HNSCC database. False discovery rate (FDR) q < 0.25 and p < 0.05 were considered statistically significant.

### Statistical analysis

Statistical analysis was performed by Bioconductor packages in R software, version 4.0.2. The prognostic ability of the derived prognostic signatures for HNSCC in comparison to other clinicopathological signatures was evaluated by receiver operating characteristic (ROC) curve analysis^22^. The independent prognostic value of the clinical characteristics for OS was evaluated by univariate and multivariate Cox proportional hazard regression analyses. The Kaplan–Meier method was used to assess the survival analysis of HNSCC patients based on the m6A-related lncRNA signature. Statistical significance was set at P < 0.05 for each analysis. The “limma R” package in R software was used for differential analysis, whereas the “ConsensusClusterPlus R”, CIBERSORT" and "ESTIMATE" R were adopted for immune infiltration analyses. The prediction model was constructed and applied in Kaplan–Meier and univariate Cox regression analysis. Besides, the “timeROC R,” “survival R,” and “glmnet R,” packages were adopted to validate the prognostic model in HNSCC.

## Results

### Construction of m6A-related lncRNA clusters

**Figure 1:**
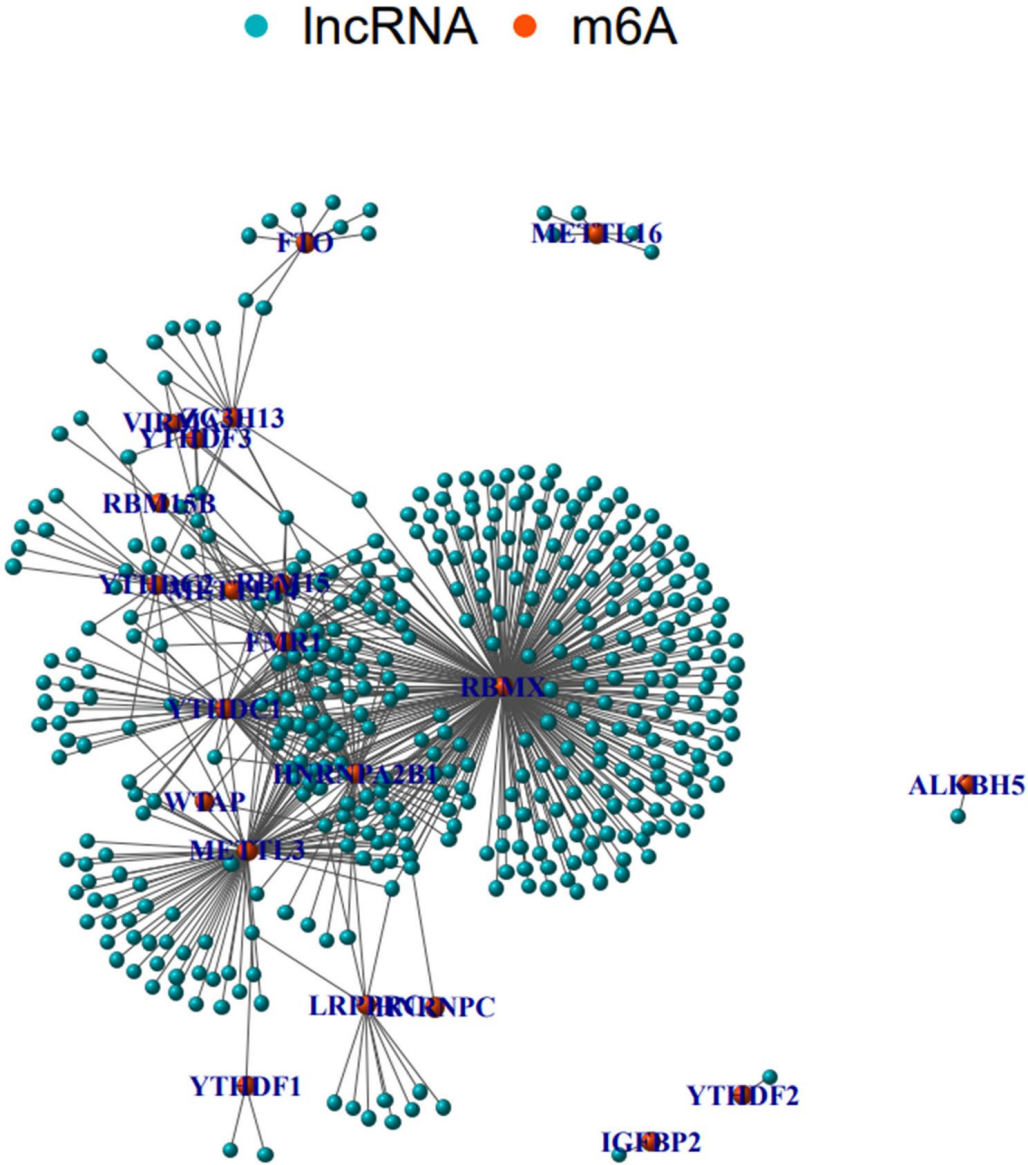
The coexpression network map between m6A and lncRNAs in HNSCC. Blue represents lncRNA, and red represents coexpressed m6A. lncRNAs, long noncoding RNA.

**Figure 2:**
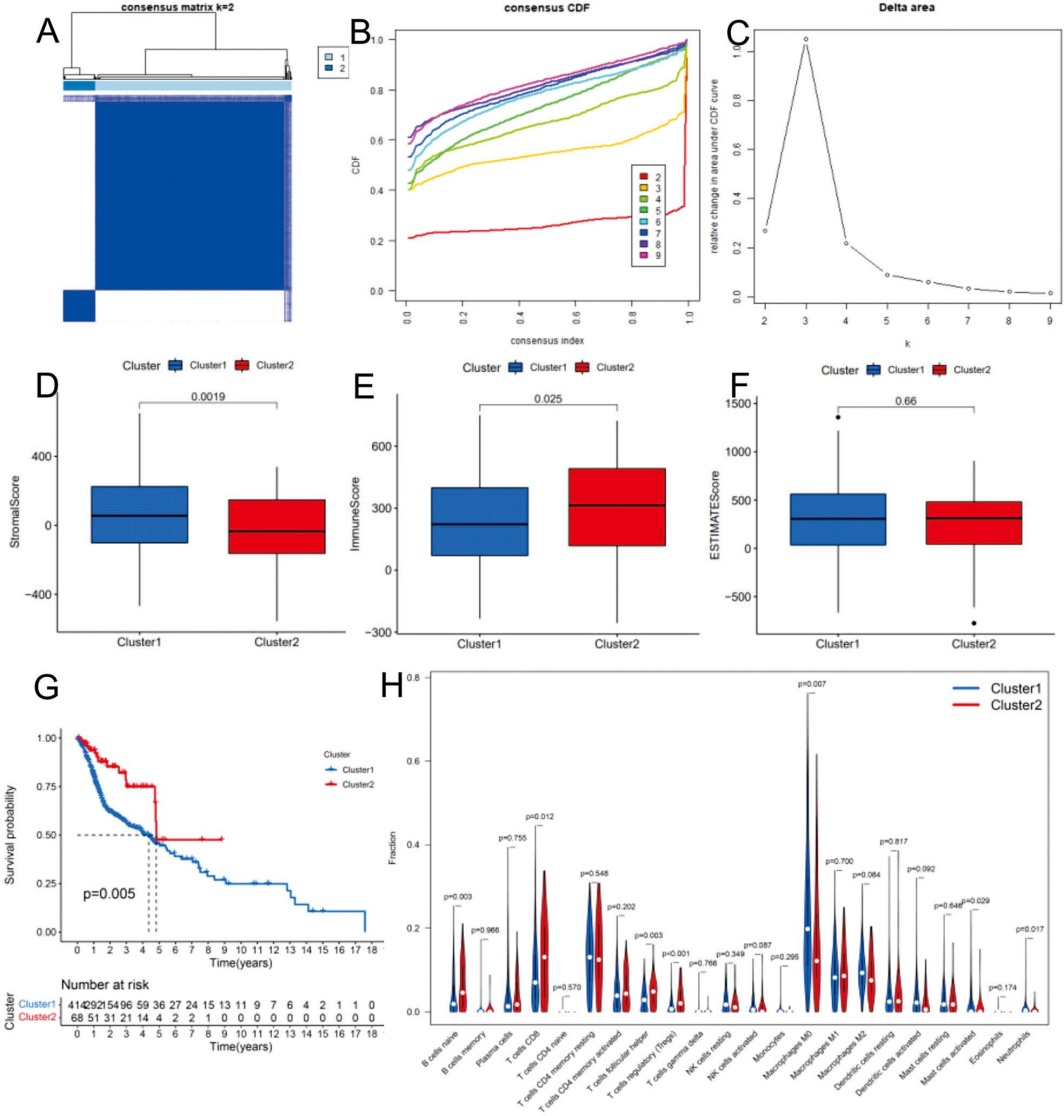
Prognosis and immune infiltrations in consensus clustering subgroups of HNSCC. (**A**) Consensus clustering matrix for k = 2. (**B**) Consensus clustering of the cumulative distribution function for k = 2–9. (**C**) Expression pattern of m6A regulators in the Cluster 1 and Cluster 2 subgroups. (**D**–**F**) Stroma, immune, and ESTIMATE scores in the Cluster 1 and Cluster 2 subgroups. (**G**) Kaplan–Meier analysis of patients in the Cluster 1 and Cluster 2 subgroups. (**H**) Immune infiltration analyses of the two m6A-related clusters. The differential proportion of 22 immune cells between the Cluster 1 and Cluster 2 subgroups. *p < 0.05; **p < 0.01.

Figure 1 shows a coexpression network map between m6A and lncRNAs in HNSCC. Blue represents lncRNA, and red represents m6A. Twenty-one m6A-related lncRNAs were mapped to the expression profile of HNSCC samples to perform consistent clustering by the CCP tool so that we could separate HNSCC samples into different tumor clusters by their different immune phenotypes. We set 9 as the maximum number of clusters (Fig. 2A). The most stable results divided the different tumor clusters into two clusters, as shown by CCP analysis, named Cluster 1 and Cluster 2 (Fig. 2B,C). We used 487 HNSCC samples from the TCGA dataset as a training group to investigate the prognosis of m6A-related lncRNA clusters. All patients in the training group were divided into Cluster 1 and Cluster 2 according to the m6A-related lncRNAs. The immune score, stromal score and tumor purity (estimated score) were all calculated by the ESTIMATE algorithm according to the gene expression profile data of 487 HNSCC samples. Then, we analyzed the disparities of the stromal score, immune score, and tumor purity (estimated score) in different m6A-related lncRNA clusters. Cluster 1 had a lower immune score and higher stromal score than Cluster 2 (Fig. 2E, P = 0.025; Fig. 2D, P = 0.0019). Similarly, Cluster 2 also had a lower tumor purity with no statistical significance (Fig. 2F, P = 0.66). Cluster 1 had a shorter overall survival (OS) than Cluster 2, as shown by Kaplan–Meier survival curves (Fig. 2G, P = 0.005). The differentiation ratio of each of 22 tumor immune cells between Cluster 1 and Cluster 2 is shown by a violin plot. The Wilcoxon rank-sum test was applied to determine significance (Fig. 2H).

### Comparison of the composition and immune cell infiltration of the TME (tumor microenvironment) in different m6A-related lncRNA clusters

**Figure 3:**
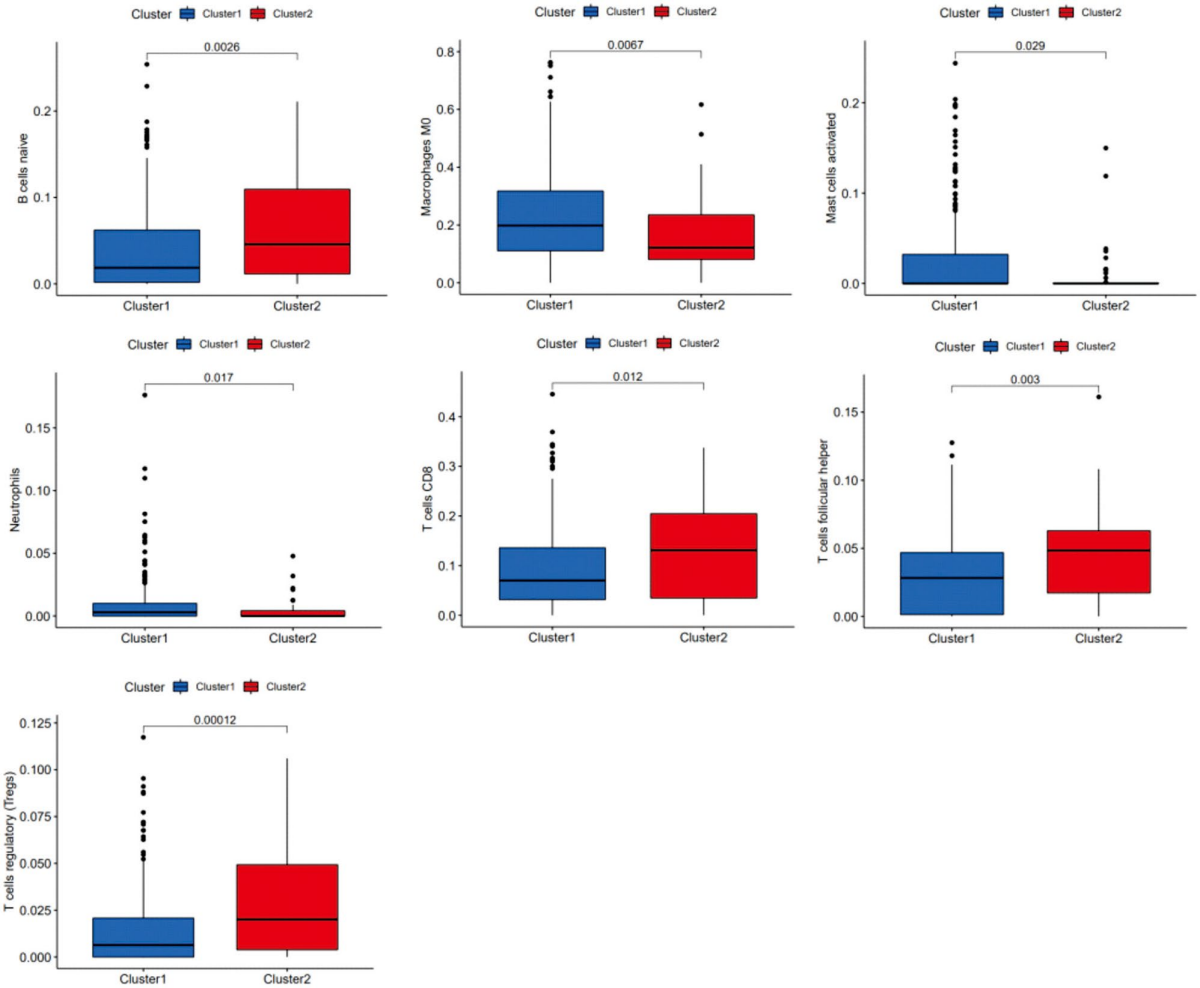
Comparison of immune cell infiltration (naive B cells, M0 macrophages, activated mast cells, neutrophils, CD8 T cells, follicular helper T cells and regulatory T cells).

The immune cell composition of 487 HNSCC samples was estimated by the CIBERSORT method. Furthermore, we normalized the results to relative proportions by cell type. Differences were observed for 7 types of immune cells. In Cluster 1, the relative levels of immune cell types were higher than those in Cluster 2, such as M0 macrophages (P = 0.0067), activated mast cells (P = 0.029), and neutrophils (P = 0.017), unlike naive B cells (P = 0.0026), CD8 T cells (P = 0.012), follicular helper T cells (P = 0.003) and regulatory T cells (Tregs) (P = 0.00012), which were lower in Cluster 1 (Fig. 3).

### Gene set enrichment analysis

**Figure 4:**
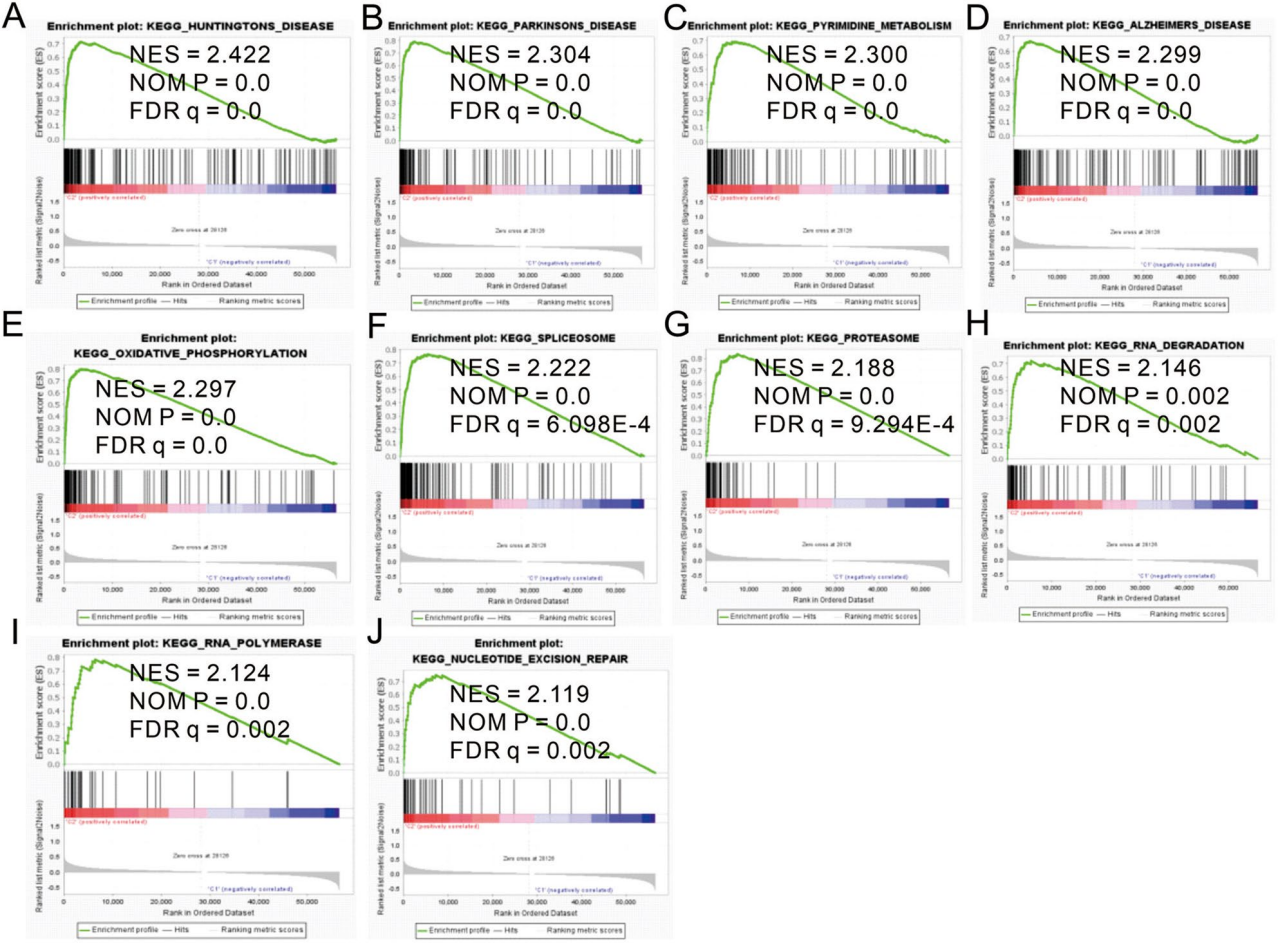
Gene set enrichment analysis performed using KEGG. |NES| > 1, NOM p < 0.05, and FDR q < 0.25 were set as the significant thresholds.

In view of the correlation between cluster and HNSCC prognosis, we conducted GSEA between Cluster 1 and Cluster 2. GSEA revealed that the majority of the novel m6A-related lncRNA prognostic signatures were positively correlated in the Cluster 2 group, including Huntington’s disease, Parkinson’s disease, pyrimidine metabolism, Alzheimer’s disease, oxidative phosphorylation, spliceosome, proteasome, RNA degradation, RNA polymerase and nucleotide excision repair (Fig. 4A–J).

### Prognostic analysis of risk model and m6A-related lncRNAs

**Figure 5:**
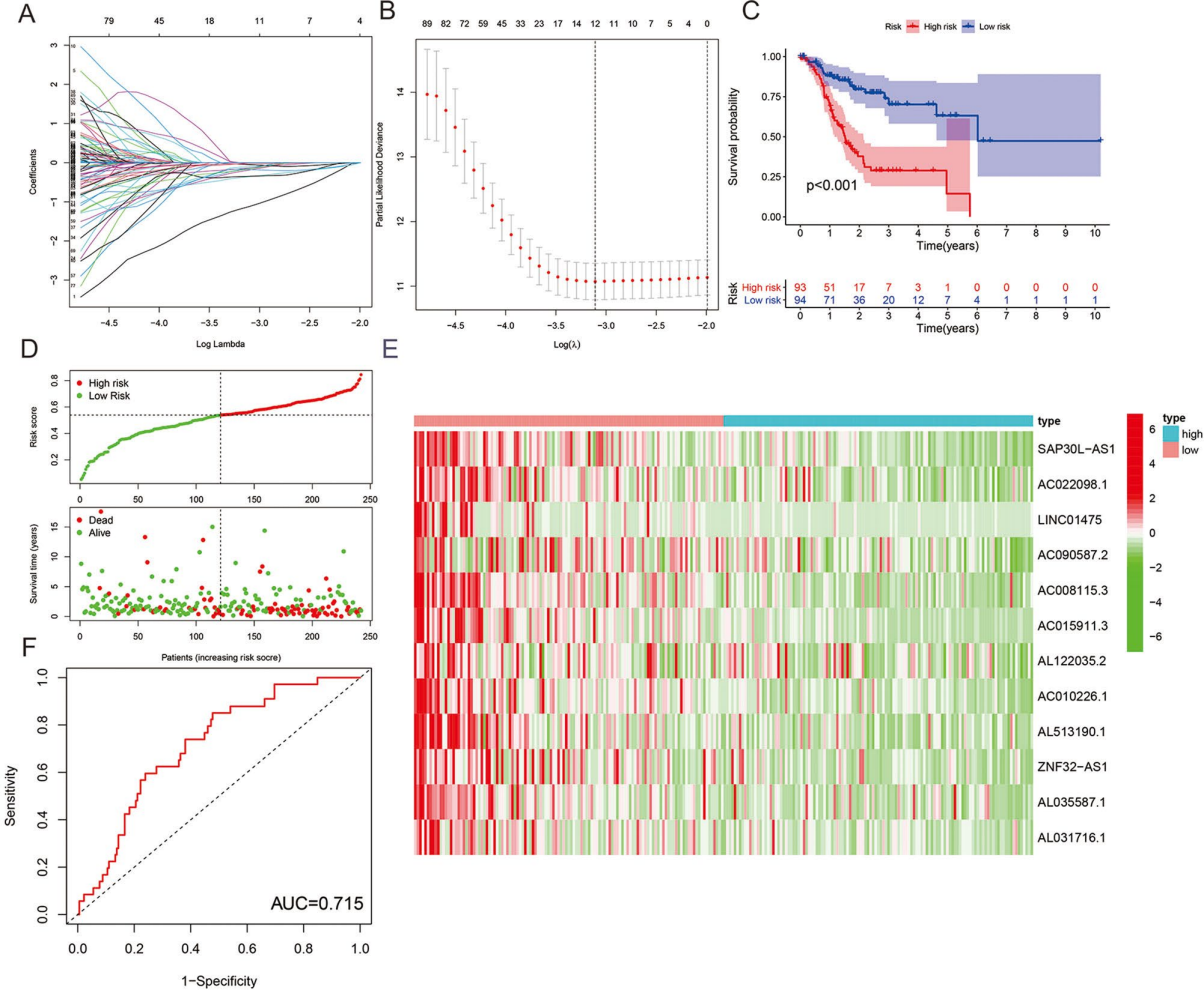
Risk model of m6A-related lncRNAs. (**A**,**B**) LASSO Cox regression analysis of 12 m6A-related genes. (**C**) Overall survival analysis of patients at high/low risk. (**D**) Risk score of each patient in the training cohort. (**E**) Expression pattern of twelve candidate m6A-related lncRNAs in the high-risk and low-risk groups. (**F**) Time-dependent ROC analysis of the risk score in predicting survival.

We developed a signature for predicting the outcomes of HNSCC by performing LASSO Cox regression analysis on 21 m6A-related lncRNAs based on the TCGA database. We obtained twelve genes (SAP30L-AS1, AC022098.1, LINC01475, AC090587.2, AC008115.3, AC015911.3, AL122035.2, AC010226.1, AL513190.1, ZNF32-AS1, AL035587.1 and AL031716.1) to build the risk model, and the risk score was calculated by the coefficients of these genes (Fig. 5A). Based on the median cutoff value of the risk score, patients with HNSCC were separated into low-risk and high-risk groups. As shown in Fig. 5B,C, Kaplan–Meier survival curves indicated that high-risk patients had significantly worse OS than low-risk patients as the risk score increased (P < 0.01). The heatmap (Fig. 5E) shows that the expression of all twelve genes decreased with an increasing risk score. The risk score and survival status distributions are plotted in Fig. 5D. The ROC curve shows that the risk score has strong predictive ability, with an AUC of 0.715 in the training set (Fig. 5F). Our results demonstrated that the risk model might serve as an important indicator for evaluating the prognosis of HNSCC patients.

### Stratification analysis of the m6A-related lncRNAs

**Figure 6:**
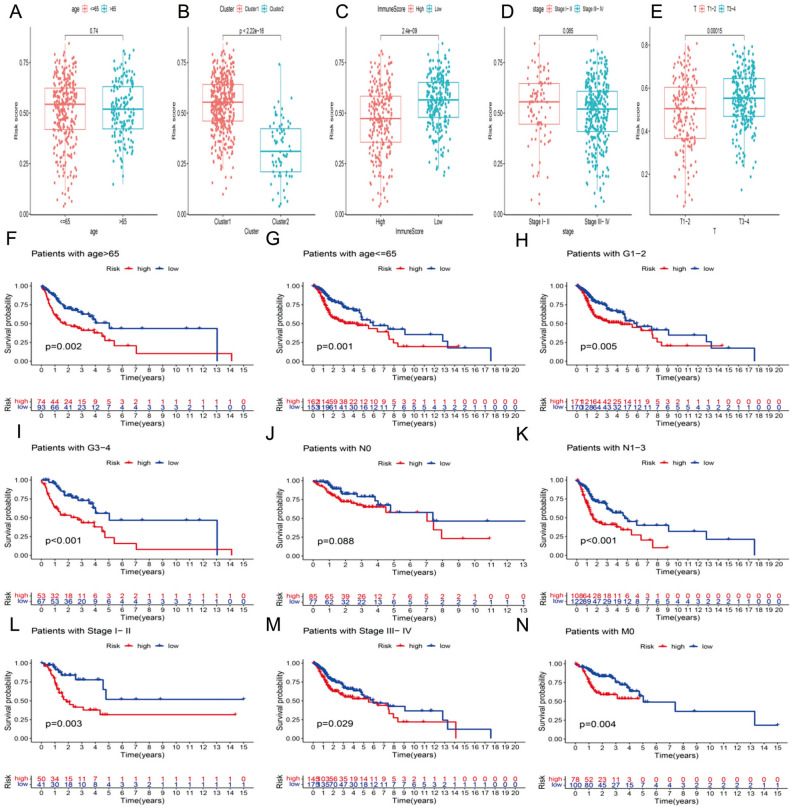
The m6A-related lncRNAs retained their prognostic value in multiple subgroups of HNSCC patients.

We explored the association between clinicopathological features and the risk score. The results revealed that HNSCC patients with Cluster 1, low immune scores, and T3-4 stage disease (Fig. 6B–D) had higher risk scores, while the risk score was not associated with age or clinical stage (Fig. 6A,E). We performed a stratification analysis to confirm whether the risk score retains its ability to predict OS in various subgroups. Our results showed that higher-risk HNSCC patients had worse OS in patients aged ≤ 65 or > 65 years, patients with G1-2 or G3-4 and patients with M0 (Fig. 6F–I,N). Moreover, the m6A-related lncRNA risk signature could distinguish the survival difference between HNSCC patients with N0 disease and patients with stage I-II disease (Fig. 6J–M).

### Effects of the risk score and clinicopathological variables on the prognosis of HNSCC patients

**Figure 7:**
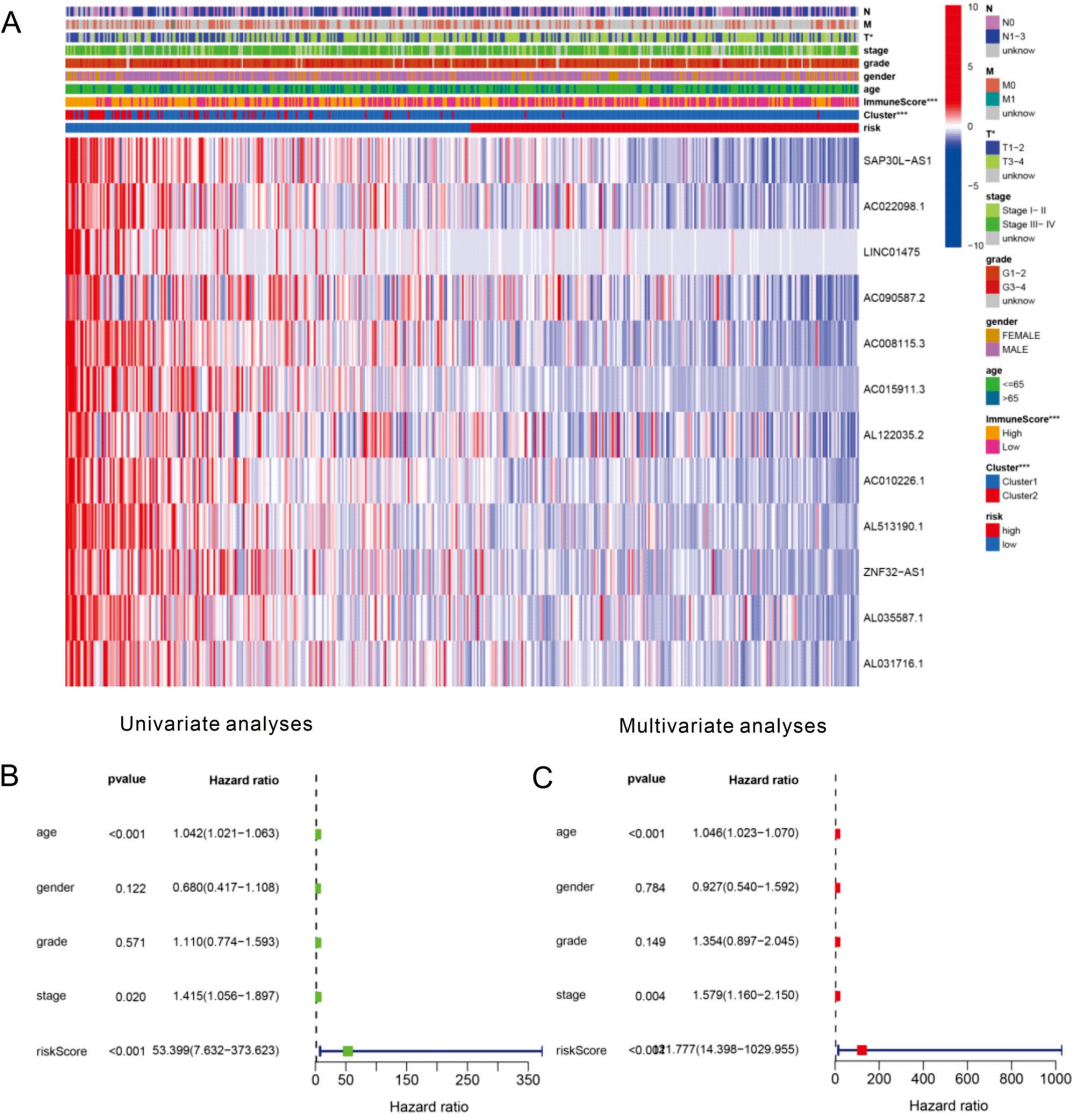
The relationship between clinicopathological features and overall survival of patients. The heatmap shows the expression of twelve m6A-related lncRNAs and the distribution of clinicopathological variables between the high- and low-risk groups. (**A**) The relative risk (HR) values of clinical features in the high-risk group by univariate and multivariate regression analysis. The vertical line is invalid. The horizontal lines in the color module represent the confidence interval of each factor (**B**,**C**).

The expression of the twelve selected m6A-related lncRNAs and clinicopathological variables in the high- and low-risk groups are shown in the heatmap. We found significant differences between the two groups in terms of immune score (P < 0.05) and cluster (P < 0.05) (Fig. 7A). Age, risk score and stage were found to be significantly linked with OS by univariate analyses (P < 0.05, Fig. 7B). The same results were observed by multivariate analyses (P < 0.05, Fig. 7C). These results suggest that the risk signature is a risk factor for HNSCC patients and can be an independent prognostic biomarker for these patients.

### Risk score associated with immune infiltration

**Figure 8:**
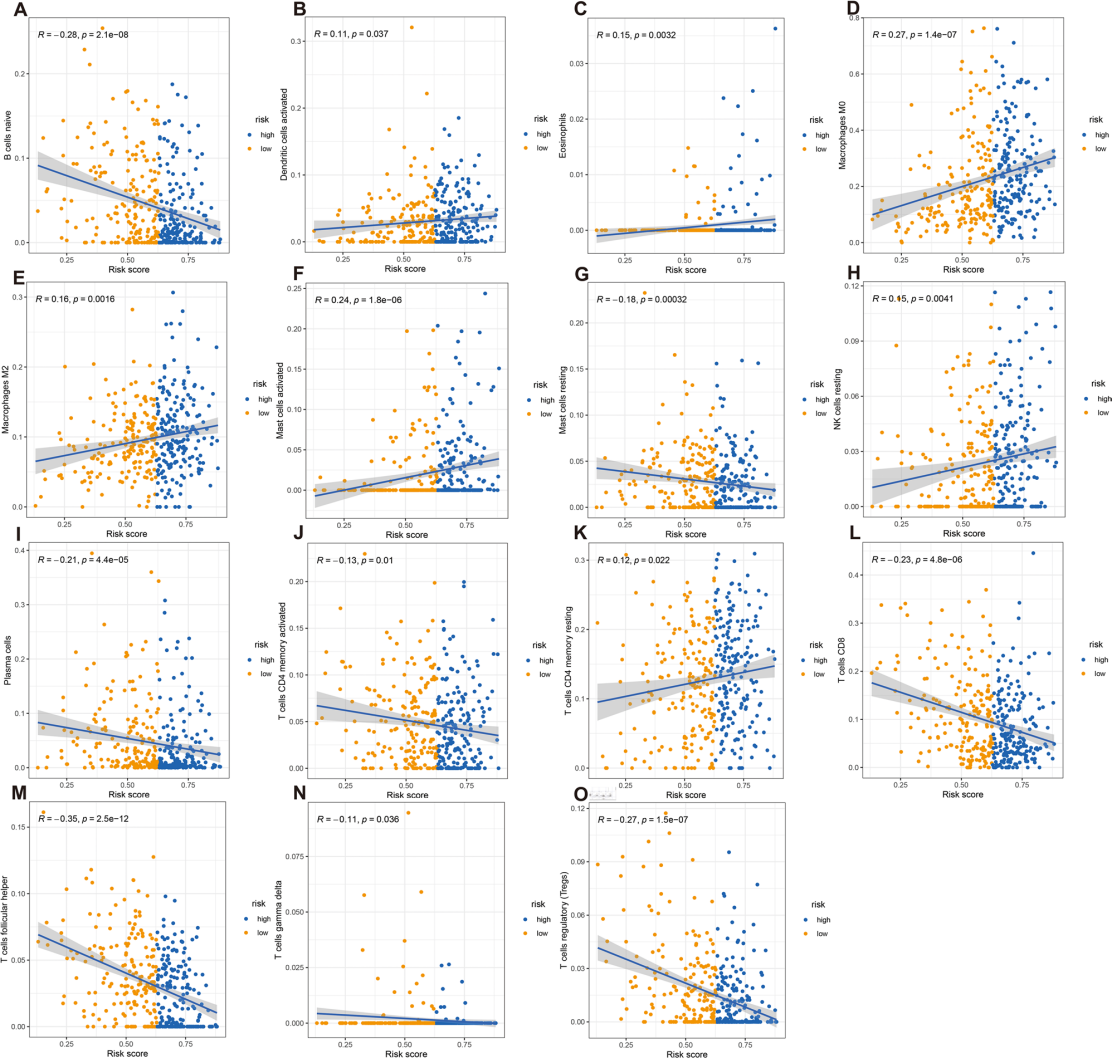
Relationship between TICs and the gene signature risk score. The blue line in each graph fits a linear model that indicates the proportional trend of the TICs and the risk score. The gray shading around the blue line indicates the 95% confidence interval. Correlation tests were conducted with Spearman’s coefficient.

Comprehensive analysis of the results of the Spearman's correlation analyses (Fig. 8A–O) showed that eosinophils, activated dendritic cells, activated mast cells, M0 macrophages, M2 macrophages, resting NK cells and resting CD4 memory T cells specifically had positive correlations with the risk score, and naive B cells, plasma cells, CD8 T cells, activated CD4 memory T cells, regulatory T cells (Tregs), follicular helper T cells, gamma delta T cells and resting mast cells had negative correlations with the risk score.

## Discussion

The treatment of HNSCC is a substantial clinical challenge due to the high rates of locoregional recurrence and chemoresistance. Furthermore, the current TNM classification system remains inapplicable for predicting HNSCC survival outcomes. It is essential to explore new biomarkers for HNSCC. However, current research on HNSCC-related biomarkers is insufficient to meet the clinical requirements for the diagnosis and prognosis of HNSCC. m6A is a potential biomarker in vivo and has been reported to be associated with various important physiological processes, such as the DNA damage response, circadian periods and stem cell differentiation^23,24^. m6A modification of lncRNAs plays key roles in oncogenesis regulation and tumor progression in various cancers. Little is known about the use of m6A-related lncRNAs as biomarkers and their internal interactions in HNSCC; therefore, it is necessary to conduct in-depth research on the influence of m6A-related lncRNAs on HNSCC.

Our study aimed to explore the potential prognostic significance of m6A-related lncRNAs using 511 HNSCC patients from TCGA. We found twelve m6A-related lncRNAs to be independent prognostic factors of HNSCC in the training cohort. HNSCC patients were divided into low-risk subgroups based on the median risk score, and high-risk patients had significantly worse OS than low-risk patients. Age, risk score and stage were found to be significantly associated with OS by Cox regression analysis. Two m6A-related lncRNA clusters with significant differences in genomic alterations and the immune microenvironment were constructed.

Infiltrating immune cells are a key component of the TME and play an important role in TME shaping^25,26^. Numerous researchers have reported how the functional status of immune cells is modulated by lncRNAs during immune responses^27–30^. The composition and immune cell types of the TME in different m6A-related lncRNA clusters were analyzed. We found that Cluster 1 had a lower immune score and higher stromal score than Cluster 2. Similarly, Cluster 1 also had higher tumor purity with no statistical significance. Kaplan–Meier survival curves showed that Cluster 2 had longer overall survival (OS) than Cluster 1.

Furthermore, differences were found in seven types of immune cells. The relative levels of immune cell types in Cluster 1, including M0 macrophages, activated mast cells, and neutrophils, were higher than those in Cluster 2, while naive B cells, follicular helper T cells, CD8 T cells and regulatory T cells (Tregs) were lower in Cluster 1. These seven types of immune cells are thought to be involved in regulating innate and adaptive immune responses and play key roles in the antitumor immune response. Macrophages play a suppressive role in the immune response^31,32^. Our results showed that Cluster 1 had a lower immune score, and the proportion of M0 macrophages was relatively high, indicating that Cluster 1 has a stronger immunosuppressive response in HNSCC samples.

Previous studies have reported the association between several of the twelve lncRNAs and cancer progression. However, there have been very few reports on the prognostic value of m6A-related lncRNAs in HNSCC patients. Thus, our study aimed to identify prognostic m6A-related lncRNAs, thereby exploring their potential roles in HNSCC tumorigenesis and progression.

In conclusion, this is the first study to identify m6A-related lncRNAs that can predict the outcomes of HNSCC. The different m6A-related lncRNAs play key roles in the complexity and heterogeneity of the TME. A twelve-gene signature model was developed that could predict the clinical progression of HNSCC. Moreover, two immune-related and prognostic m6A-related lncRNA clusters were constructed with significant differences in genomic alterations and the immune microenvironment in HNSCC samples. The m6A-related lncRNA clustering analysis of HNSCC samples provides new insight into the stratification of patients with different immune phenotypes as well as an in-depth understanding of immune molecular mechanisms in HNSCC. This study also has certain limitations. First, we analyzed m6A-related lncRNAs and genetic data only in HNSCC samples without normal controls. Second, this study is a bioinformatic and retrospective study. Third, we did not verify the lncRNA signature on other datasets.

## References

[1] Siegel R (2014). Cancer statistics, 2014. CA Cancer J. Clin..

[2] Leemans CR, Braakhuis BJ, Brakenhoff RH (2011). The molecular biology of head and neck cancer. Nat. Rev. Cancer.

[3] Shah I (2010). Clinical stage of oral cancer patients at the time of initial diagnosis. J. Ayub Med. Coll. Abbottabad.

[4] Warnakulasuriya S (2009). Global epidemiology of oral and oropharyngeal cancer. Oral Oncol..

[5] Takes RP (2012). Distant metastases from head and neck squamous cell carcinoma. Part I. Basic aspects. Oral Oncol..

[6] Conley BA (2006). Treatment of advanced head and neck cancer: What lessons have we learned?. J. Clin. Oncol..

[7] Noorlag R (2015). Nodal metastasis and survival in oral cancer: Association with protein expression of SLPI, not with LCN2, TACSTD2, or THBS2. Head Neck.

[8] Alarcón CR (2015). N6-methyladenosine marks primary microRNAs for processing. Nature.

[9] Chen XY, Zhang J, Zhu JS (2019). The role of m(6)A RNA methylation in human cancer. Mol. Cancer.

[10] Hong K (2018). Emerging function of N6-methyladenosine in cancer. Oncol. Lett..

[11] Wahlestedt C (2013). Targeting long non-coding RNA to therapeutically upregulate gene expression. Nat. Rev. Drug Discov..

[12] Matouk IJ (2007). The H19 non-coding RNA is essential for human tumor growth. PLoS ONE.

[13] Zhou Y, Zhang X, Klibanski A (2012). MEG3 noncoding RNA: A tumor suppressor. J. Mol. Endocrinol..

[14] Dai D (2018). N6-methyladenosine links RNA metabolism to cancer progression. Cell Death Dis..

[15] Ban Y (2020). LNCAROD is stabilized by m6A methylation and promotes cancer progression via forming a ternary complex with HSPA1A and YBX1 in head and neck squamous cell carcinoma. Mol. Oncol..

[16] Zhong J (2013). EZH2 regulates the expression of p16 in the nasopharyngeal cancer cells. Technol. Cancer Res. Treat..

[17] Wu KJ (2020). The role of miRNA biogenesis and DDX17 in tumorigenesis and cancer stemness. Biomed. J..

[18] Wojtas MN (2017). Regulation of m(6)A transcripts by the 3′→5′ RNA helicase YTHDC2 is essential for a successful meiotic program in the mammalian germline. Mol. Cell.

[19] Tang C (2018). ALKBH5-dependent m6A demethylation controls splicing and stability of long 3'-UTR mRNAs in male germ cells. Proc. Natl. Acad. Sci. USA.

[20] Yoshihara K (2013). Inferring tumour purity and stromal and immune cell admixture from expression data. Nat. Commun..

[21] Chen B (2018). Profiling tumor infiltrating immune cells with CIBERSORT. Methods Mol. Biol..

[22] Heagerty PJ, Lumley T, Pepe MS (2000). Time-dependent ROC curves for censored survival data and a diagnostic marker. Biometrics.

[23] Geula S (2015). Stem cells. m6A mRNA methylation facilitates resolution of naïve pluripotency toward differentiation. Science.

[24] Xiang Y (2017). RNA m(6)A methylation regulates the ultraviolet-induced DNA damage response. Nature.

[25] Binnewies M (2018). Understanding the tumor immune microenvironment (TIME) for effective therapy. Nat. Med..

[26] Roma-Rodrigues C (2019). Targeting tumor microenvironment for cancer therapy. Int. J. Mol. Sci..

[27] Chen YG, Satpathy AT, Chang HY (2017). Gene regulation in the immune system by long noncoding RNAs. Nat. Immunol..

[28] Jiang R (2017). The long noncoding RNA lnc-EGFR stimulates T-regulatory cells differentiation thus promoting hepatocellular carcinoma immune evasion. Nat. Commun..

[29] Chen C (2018). LNMAT1 promotes lymphatic metastasis of bladder cancer via CCL2 dependent macrophage recruitment. Nat. Commun..

[30] Li Z, Rana TM (2014). Decoding the noncoding: Prospective of lncRNA-mediated innate immune regulation. RNA Biol..

[31] Gambardella V (2020). The role of tumor-associated macrophages in gastric cancer development and their potential as a therapeutic target. Cancer Treat. Rev..

[32] Capece D (2013). The inflammatory microenvironment in hepatocellular carcinoma: A pivotal role for tumor-associated macrophages. Biomed. Res. Int..

